# Supplementary material for: Identification of photoperiod-induced specific miRNAs in the adrenal glands of Sunite sheep (Ovis aries)
Source: Front Vet Sci. 2022 Jul 22;9:888207. doi: 10.3389/fvets.2022.888207 (PMC9354845; doi:10.3389/fvets.2022.888207)
Supplement: Supplementary file 1 [file Data_Sheet_1.ZIP › Supplementary file/miRDeep2 file.zip!/miRDeep2 file/Novel_Structure/SPLP42A3/Novel_533.pdf]

Provisional ID	:	NC_019477.2_31710
Score total	:	3.4
Score for star read(s)	:	3.9
Score for read counts	:	-3.9
Score for mfe	:	1.8
Score for randfold	:	1.6
Score for cons. seed	:	
Total read count	:	4
Mature read count	:	2
Loop read count	:	0
Star read count	:	2

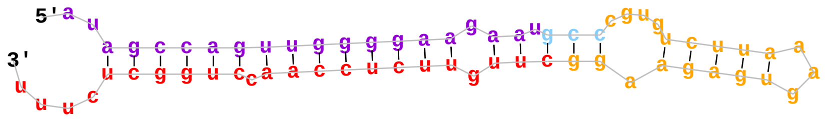

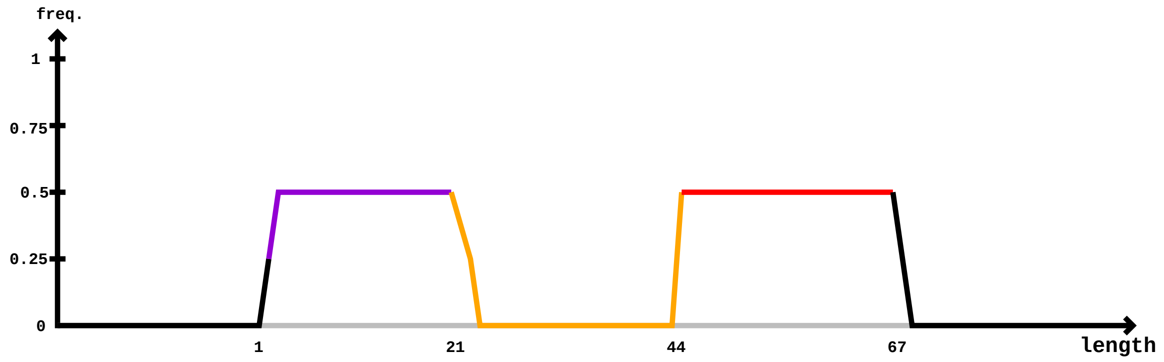


```
                   Star                                        Mature
5'- aaggcuaauccuauugaggcauagccaguuggggaagaaugcccgugucuuaaagugagaaggcuuguucuccaaccuggcucuuuauggcugcuggggaaaggcaugaa  -3'   obs
    aaggcuaauccuauugaggcauagccaguuggggaagaaugcccgugucuuaaagugagaaggcuuguucuccaaccuggcucuuuauggcugcuggggaaaggcaugaa        exp
    ...(((..((((....((.(((((((((((((((((.((.(((....(((((...))))).))))).)))))))).))))))....))).))....))))..))).....  reads   mm   sample
    ....................auagccaguuggggaagaau......................................................................  1       0    seq
    .....................uagccaguuggggaagaaugc....................................................................  1       0    seq
    ...............................................................cuuguucuccaaccuggcucuuu........................  1       0    seq
    ...............................................................cuuguucuccaaccuggcucuuua.......................  1       0    seq
```
